# Supplementary material for: S2DB-mmWave YOLOv8n: Multi-object detection for millimeter-wave radar using YOLOv8n with optimized multi-scale features
Source: PLoS One. 2025 Sep 19;20(9):e0332931. doi: 10.1371/journal.pone.0332931 (PMC12448986; doi:10.1371/journal.pone.0332931)
Supplement: Attachment — Submitted filename: Response to Reviewers.docx [file pone.0332931.s002.docx]

**Response to Reviewers**

Dear Dr. Aunsri and Reviewers,

Thank you for your correspondence and the reviewers’ constructive comments regarding our manuscript titled “S2DB-mmWave YOLOv8n: Multi-Object Detection for Millimeter-Wave Radar Using YOLOv8n with Optimized Multi-Scale Features”(Manuscript ID: PONE-D-25-21299). We sincerely appreciate the time and expertise dedicated to evaluating our work, which has provided valuable insights for strengthening the rigor and clarity of this research.

In this revised manuscript and the accompanying point-by-point response document (uploaded separately), we have carefully systematically all suggestions. Significant revisions include:

(1) In Section 1, we have provided a detailed analysis of the strengths and limitations of the existing technology to offer a comprehensive overview. Additionally, we have clearly listed the main contributions of this study to highlight its significance and innovations.
(2) The details of the model have been elaborated more clearly in Section 2.2. We added feature visualization images(Fig8(b)). These additions enable a more accurate evaluation of the performance enhancement achieved by integrating the module.

(3) Added GFLOPs and FPS to the evaluation metrics section in Section 3.3, and included an analysis of these results in the experiments presented in Section 3.4, and we added comparative analysis with state-of-the-art models (YOLOv12), making the model assessment more comprehensive.

We confirm that every reviewer comment has been thoroughly addressed with corresponding modifications in the manuscript. The track-changes version and clean copy are provided for your convenience.

**Academic Editor:**

1) ***.The paper proposes a target detection and classification framework of S2DB-mmWave YOLOv8n, based on deep learning aiming to gain more accuracy. The authors are suggested to provide a table to list pros and cons of the existing works and then this would lead to the importance of this work. Therefore, list of contributions must be provided. Also, the complexity analysis of the proposed method with comparison to the other state-of-the-art has not been provided in the paper, this must be presented.***

***(1)The authors are suggested to provide a table to list pros and cons of the existing works.***

**Response:** Thank you for your valuable comments. I have carefully considered your suggestions and made the following revisions to enhance the clarity and completeness of our manuscript. I Added a detailed analysis of the strengths and weaknesses of existing methods at line 140 in the introduction section. This provides a more comprehensive context for our work.

Revised Section:

Line138-Line139:

The advantages and disadvantages of the aforementioned related methods are listed in Table 1.

Table1:

**Table 1. Advantages and Disadvantages Analysis of Related Methods.**

| Methods | Advantages | Disadvantages |
| --- | --- | --- |
| CFAR | Strong adaptability and Real-time capability | Not suitable for non-uniform backgrounds andLacks contextual understanding |
| [15-16] | Simplifies detection workflow and validates CNN effectiveness | Simulated data only, not validated in real-world scenarios |
| [17] | Achieves wide-angle 180° panoramic observation through radar rotation | Larger errors for small targets or distant targets |
| [18] | Improves detection accuracy after denoising | Denoising suppresses large/small targets and potential information loss |
| [19] | High-quality radar imaging, suitable for fusion with RGB data | Privacy concerns and poor detection of low-resolution small targets |
| [20] | More accurate recognition of large targets like vehicles | Confusion in direction judgment when target speed is slow or point cloud is sparse |
| [21] | Robust detection without background suppression | Validation focused on single-target; multi-object detection unverified |
| [22] | Combination of 3D and 2D detection heads | Not yet supported for real-time embedded deployment; requires optimization and lightweight structure |
| [23] | Adaptable to different environments and scenarios | Requires significant computational resources to handle multimodal data |

***(2)list of contributions must be provided.***

**Response:** Thank you for your valuable comments. The main contributions of this study are now clearly listed at line 146 in the introduction section. This summary helps readers quickly grasp the key innovations and advancements presented in our research.

Revised Section:

Line146-Line164:

The principal contributions of this work can be summarized as follows:

1. Advanced convolutional layers integrated with a Simplified Spatial Pyramid Pooling - Fast (SimSPPF) structure are introduced into the backbone network, replacing conventional convolutional operations and the Spatial Pyramid Pooling - Fast(SPPF) module. This configuration mitigates gradient vanishing while reducing information loss, thereby significantly enhancing both feature extraction capabilities and network robustness—particularly when processing low-resolution radar heatmap data.

2. To mitigate the loss of fine-grained information during upsampling in YOLOv8n, we propose a novel upsampling module. By adaptively fusing multi-level feature representations, this design enables more precise image detail reconstruction and substantially improves object localization accuracy.

3. The neck network incorporates a BiFPN module to optimize multi-scale feature fusion, thereby improving detection performance for objects at varying scales.

4. The proposed S2DB-mmWave YOLOv8n model achieves 93.1% mAP@0.5 and 55.8% mAP@0.5:0.95 on the mmWave radar range-azimuth heatmap dataset, surpassing the baseline by 3.3% and 1.6% respectively, with concurrent precision and recall rates of 89.4% and 90.6%, which demonstrates significant improvements in classification accuracy and overall detection performance for mmWave radar systems.

***(3)Also, the complexity analysis of the proposed method with comparison to the other state-of-the-art has not been provided in the paper, this must be presented.***

**Response:** Thank you for your valuable comments. I introduced GFLOPs and FPS at line 354 in the evaluation metrics section to provide a more holistic assessment of our model’s performance. Additionally, I have included data comparisons in Table 4 at line 372 and Table 5 at line 398, along with corresponding analyses at line 382 and line 422. To further validate our model’s performance, we have also added a comparative experiment with the latest object detection model, YOLOv12 in Table4. This comparison provides a benchmark against state-of-the-art techniques and demonstrates the competitive edge of our proposed approach. These additions offer a more thorough evaluation and facilitate a better understanding of our model’s efficiency and effectiveness.

Revised Section:

Line354-Line364:

This paper uses frames per second (FPS) to evaluate the inference speed of the model. The core idea behind calculating FPS is to measure the total time required for the model to process a certain number of images, and then divide the number of images by this total time to obtain the number of image frames processed per second,as shown in Equation (10).

$FPS=\frac{n(\text{ image })}{t}$	(10)

The Giga Floating Point Operations per Second (GFLOPs) is a metric for measuring computational performance, indicating the number of billions of floating-point operations that can be executed per second. When describing model performance, GFLOPs is commonly used to assess the computational complexity and efficiency of a model when processing data.

Table4:

**Table 4. Comparative test results.**

| Model | mAP @0.5  （%） | mAP @0.5:0.95  （%） | Precision  （%） | Recall  （%） | Parameters  （M) | FPS | GFLOPS |
| --- | --- | --- | --- | --- | --- | --- | --- |
| RT-DETR | 89.9 | 53.5 | 89.2 | 85.4 | 31.3 | 90.0 | 103.5 |
| YOLOv5n | 88.1 | 51.0 | 79.6 | 83.9 | 2.6 | **125.1** | 7.1 |
| YOLOv6n | 85.1 | 47.5 | 74.2 | 81.5 | 4.2 | 85.3 | 9.8 |
| YOLOv7-PM | 90.1 | 49.5 | 89.2 | 84.1 | 71.5 | 81.9 | 189 |
| PiX2PiX  +YOLOv7-PM | 91.8 | 52.5 | **92.4** | 83.9 | 92.3 | 75.6 | 231.2 |
| YOLOv9-c | 86.9 | 47.4 | 88.0 | 76.9 | 25.3 | 35.0 | 103.6 |
| YOLOv11n | 91.1 | 56.3 | 84.3 | 83.9 | 2.6 | 82.0 | 6.3 |
| YOLOv12n | 90.5 | 54.9 | 87.5 | 83.0 | **2.5** | 99.1 | **5.8** |
| YOLOv8n  -Baseline | 89.8 | 54.2 | 84.9 | 82.9 | 3.2 | 89.2 | 8.1 |
| **S2DB-mmWave**  **YOLOv8n** | **93.1** | **55.8** | 89.4 | **90.6** | 3.2 | 89.3 | 8.1 |

Line382-Line388:

The S2DB-mmWave YOLOv8n model achieves high accuracy with a computational complexity of 8.1 GFLOPs, positioning it in the lower-middle range among comparative baseline modes. This indicates well-preserved feature extraction capabilities without substantial computational overhead. The model attains a competitively high FPS, demonstrating real-time processing efficiency. Furthermore, its efficient architecture design ensurs favorable scalability and hardware compatibility, enabling deployment across diverse operational scenarios.

Table5:

**Table 5. Results of ablation experiments.**

| NO. | BiFPN | Dysample | Sim Backbone | mAP  @0.5  (%) | mAP  @0.5:0.95  (%) | Precision  (%) | Recall  (%) | FPS | GFLOPs |
| --- | --- | --- | --- | --- | --- | --- | --- | --- | --- |
| 1 | × | × | × | 89.8 | 54.2 | 84.9 | 82.9 | **89.3** | **8.1** |
| 2 | √ | × | × | 91.6 | 53.4 | 88.3 | 85.3 | 48.1 | 8.1 |
| 3 | × | √ | × | 90.3 | 55.1 | 83.8 | 86.3 | 89.3 | 8.1 |
| 4 | × | × | √ | 91.3 | 54 | 86.9 | 84.4 | 87.7 | 8.1 |
| 5 | √ | √ | × | 92.3 | 54.5 | 86.6 | 89.1 | 87.0 | 8.1 |
| 6 | √ | √ | √ | **93.1** | **55.8** | **89.4** | **90.6** | 89.3 | 8.1 |

Line422-Line423:

but also maintains the FPS and GFLOPs, ensuring efficiency and performance.

Table4:

**Table 4. Comparative test results.**

| Model | mAP @0.5  （%） | mAP @0.5:0.95  （%） | Precision  （%） | Recall  （%） | Parameters  （M) | FPS | GFLOPS |
| --- | --- | --- | --- | --- | --- | --- | --- |
| RT-DETR | 89.9 | 53.5 | 89.2 | 85.4 | 31.3 | 90.0 | 103.5 |
| YOLOv5n | 88.1 | 51.0 | 79.6 | 83.9 | 2.6 | **125.1** | 7.1 |
| YOLOv6n | 85.1 | 47.5 | 74.2 | 81.5 | 4.2 | 85.3 | 9.8 |
| YOLOv7-PM | 90.1 | 49.5 | 89.2 | 84.1 | 71.5 | 81.9 | 189 |
| PiX2PiX  +YOLOv7-PM | 91.8 | 52.5 | **92.4** | 83.9 | 92.3 | 75.6 | 231.2 |
| YOLOv9-c | 86.9 | 47.4 | 88.0 | 76.9 | 25.3 | 35.0 | 103.6 |
| YOLOv11n | 91.1 | 56.3 | 84.3 | 83.9 | 2.6 | 82.0 | 6.3 |
| YOLOv12n | 90.5 | 54.9 | 87.5 | 83.0 | **2.5** | 99.1 | 5.8 |
| YOLOv8n  -Baseline | 89.8 | 54.2 | 84.9 | 82.9 | 3.2 | 89.2 | 8.1 |
| **S2DB-mmWave**  **YOLOv8n** | **93.1** | **55.8** | 89.4 | **90.6** | 3.2 | 89.3 | 8.1 |

We believe these revisions significantly enhance the quality and impact of our manuscript. Thank you again for your insightful feedback.

2) ***. Please ensure that your manuscript meets PLOS ONE's style requirements, including those for file naming. The PLOS ONE style templates can be found at <https://journals.plos.org/plosone/s/file?id=wjVg/PLOSOne_formatting_sample_main_body.pdf> and <https://journals.plos.org/plosone/s/file?id=ba62/PLOSOne_formatting_sample_title_authors_affiliations.pdf>.***

**Response:** Thank you for your comment. We have reviewed the manuscript formatting according to the provided template.We have ensured that the manuscript meets PLOS ONE's requirements, including file naming requirements.

Revised Section:

Line13:

E-mail:yyjxcu@126.com(YY);dugy@xcu.edu.cn(GD)

3) ***. Please note that PLOS ONE has specific guidelines on code sharing for submissions in which author-generated code underpins the findings in the manuscript. In these cases, we expect all author-generated code to be made available without restrictions upon publication of the work. Please review our guidelines at https://journals.plos.org/plosone/s/materials-and-software-sharing#loc-sharing-code and ensure that your code is shared in a way that follows best practice and facilitates reproducibility and reuse.***

**Response:** Thank you for your comment. We've uploaded the code to github (mengqizh1010/S2DB (github.com)) and made sure it follows best practices and facilitates reproducible and reusable sharing.

4) ***. Thank you for stating the following financial disclosure:[This work was supported by Henan Province Key R&D Special Project (No. 241111212500) and Henan Province Key R&D and Promotion Special(Technology Tackling Key) Project(No.232102210181).].Please state what role the funders took in the study.  If the funders had no role, please state: ""The funders had no role in study design, data collection and analysis, decision to publish, or preparation of the manuscript.""If this statement is not correct you must amend it as needed.Please include this amended Role of Funder statement in your cover letter; we will change the online submission form on your behalf.***

**Response:** The funders had no role in study design, data collection and analysis, decision to publish, or preparation of the manuscript. Thanks.

5) ***. We note that Thank you for stating the following in the Acknowledgments Section of your manuscript:[This work was supported by Henan Province Key R&D Special Project (No. 241111212500) and Henan Province Key R&D and Promotion Special(Technology Tackling Key) Project(No.232102210181).]We note that you have provided funding information that is not currently declared in your Funding Statement. However, funding information should not appear in the Acknowledgments section or other areas of your manuscript. We will only publish funding information present in the Funding Statement section of the online submission form.Please remove any funding-related text from the manuscript and let us know how you would like to update your Funding Statement. Currently, your Funding Statement reads as follows:[This work was supported by Henan Province Key R&D Special Project (No. 241111212500) and Henan Province Key R&D and Promotion Special(Technology Tackling Key) Project(No.232102210181).].Please include your amended statements within your cover letter; we will change the online submission form on your behalf.***

**Response:** Thank you for your comment.We have revised our manuscript according to your instructions. Specifically, we have removed the funding-related text from the Acknowledgments section and updated the Funding section as follows:

Funding :

This work was supported by the Henan Province Key R&D Special Project (No. 241111212500) and the Henan Province Key R&D and Promotion Special (Technology Tackling Key) Project (No. 232102210181).

We have also included these changes in the revised manuscript. Thank you for your understanding and support.

6) ***. We note that your Data Availability Statement is currently as follows: [All relevant data are within the manuscript and its Supporting Information files.]Please confirm at this time whether or not your submission contains all raw data required to replicate the results of your study. Authors must share the “minimal data set” for their submission.***

**Response:** Thank you for your comment. We confirm that all raw data required to replicate the results of our study are available. The original dataset used in this study is publicly available and has been appropriately cited in the manuscript. Additionally, all data augmentation techniques applied to the training set, including the parameters used, are clearly described in the Methods section of the manuscript.Therefore, we believe that our submission includes the minimal data set necessary for replication, as required.Please let us know if any further clarification or additional information is needed.

**Thank you once again for your careful review and valuable attention to our work. We truly appreciate your efforts and look forward to the possibility of further collaboration in the future.**

**Reviewer #1:**

*Comments: The paper presents an interesting and relevant contribution to the field of radar-based object detection by integrating an optimized multi-scale feature enhancement strategy into the lightweight YOLOv8n framework. The following are required to be revised for the improvement of this paper.*

***1). The description of the S2DB module lacks sufficient detail regarding its internal operations and parameters. It would be helpful to include a clearer diagram and a pseudocode-style explanation of how the dual-branch mechanism selects and fuses multi-scale features.***

**Response:** Thank you for your meticulous efforts and constructive comments. We have taken your suggestions to heart and have made several improvements to enhance the clarity and comprehensiveness of our work. Specifically, we have provided a more detailed explanation of the module’s details, supplemented by pseudocode to facilitate a better understanding of the module’s functionality. Additionally, we have revised the module’s structure diagram and incorporated relevant formulas to elucidate the internal processes more transparently. We believe these enhancements will significantly improve the readability and interpretability of our manuscript.

Revised Section:

Fig2:

**Fig 2. Improved network structure of the YOLOv8n model.**

Line252-Line257:

DySample takes a low-resolution feature map χ as input and first generates a dynamic range adjustment factor through a linear transformation layer (linear1), constraining its value within [0, 0.5] to control the sampling range. Subsequently, another linear transformation layer (linear2) produces an initial offset O_init_. By combining the dynamic adjustment factor with the initial offset O_init_, a dynamic offset is generated. This offset O has dimensions

Line259-Line260:

Fig 7 displays key parts of the pseudocode within the DySample module.Fig6(b):

**Fig6(b) The structure of the sample point generator in Dysample.**

Fig7:

**Fig 7. The key parts of the pseudocode within the DySample module.**

Line286-Line295:

Using the P6-level feature map (illustrated in Fig. 8(b) as an example), BiFPN generates two fused features: the top-down aggregated feature $P_{6}^{td}$ and the output feature $P_{6}^{\text{out }}$,formally expressed as:

$P_{6}^{td}=\mathrm{Conv}\left( \frac{w_{1}\cdot P_{6}^{in}+w_{2}\cdot\mathrm{Resize}\left( P_{7}^{in} \right)}{w_{1}+w_{2}+\epsilon} \right)$	(3)

$P_{6}^{\text{out }}=\mathrm{Conv}\left( \frac{w_{1}^{'}\cdot P_{6}^{\text{in }}+w_{2}^{'}\cdot P_{6}^{td}+w_{3}^{'}\cdot\mathrm{Resize}\left( P_{5}^{\text{out }} \right)}{w_{1}^{'}+w_{2}^{'}+w_{3}^{'}+\epsilon} \right)$	(4)

where $P_{5}^{\text{out }}$, $P_{6}^{in}, P_{6}^{td}$, $P_{6}^{\text{out }}$, and $P_{7}^{in}$ denote the output feature at level 5, the input feature at level 6, the intermediate feature at level 6, the output feature at level 6, and the input feature at level 7, respectively; Conv represents the convolution operation; Resize refers to upsampling or downsampling operations; $w$ and $w^{'}$ are learnable weight parameters; and $\epsilon$ is set to 0.0001 to ensure numerical stability.

***2). It would be valuable to evaluate the impact of individual components of the feature optimization strategy (e.g., feature scaling parameters, fusion technique) to better isolate their contributions to overall performance.***

**Response:** Thank you for your comment. In the ablation study comparison within the experimental section, we have elaborated on the impact of each component of the model on its overall performance. Furthermore, to offer a clearer illustration of the module’s impact, we have incorporated a visual comparison of features before and after feature fusion in Figure 8(b). This enhancement allows for a more intuitive understanding of the module’s role in improving the model’s performance. We trust that these revisions address your concerns and provide a more comprehensive evaluation of our approach.

Revised Section:

Line274-Line276:

The use of simple addition (Add) or concatenation (Concat) does not adequately account for the relative importance of each feature during the fusion process.

Line279-Line280:

and shows the feature maps of both inputs and outputs.

Fig8(b):

**Fig8(b) BiFPN.**

***3). Please clarify whether the mmWave radar dataset used in this work is publicly available or proprietary. If it is not public, consider discussing the possibility of releasing a subset or detailed dataset specifications to enhance reproducibility.***

**Response:** Thank you for your insightful comment. We appreciate your attention to the dataset used in our study. As mentioned in the manuscript, the dataset employed is publicly available and has been properly cited. To bolster the robustness and generalization capability of our model, we applied data augmentation techniques to the training subset of this dataset. The specific parameters utilized in this process are thoroughly documented within the text. We believe these enhancements contribute significantly to the overall reliability and applicability of our model.

***4). The paper briefly mentions the computational efficiency of YOLOv8n, but it would be informative to provide precise metrics (e.g., FPS on a given hardware setup, parameter count, FLOPs) for both the baseline and the proposed model to quantify trade-offs between detection accuracy and processing speed.***

**Response:** Thank you for your careful work and kind comments. In response, we have incorporated additional data comparisons of GFLOPs and FPS into the comparative experiments and ablation studies within the experimental section, conducted under specific hardware configurations. This enhancement enables a more comprehensive and detailed evaluation of the different models, providing clearer insights into their respective performance characteristics. We believe these additions significantly strengthen the analysis and address the concerns raised.

Revised Section:

Line354-Line364:

This paper uses frames per second (FPS) to evaluate the inference speed of the model. The core idea behind calculating FPS is to measure the total time required for the model to process a certain number of images, and then divide the number of images by this total time to obtain the number of image frames processed per second,as shown in Equation (10).

$FPS=\frac{n(\text{ image })}{t}$	(10)

The Giga Floating Point Operations per Second (GFLOPs) is a metric for measuring computational performance, indicating the number of billions of floating-point operations that can be executed per second. When describing model performance, GFLOPs is commonly used to assess the computational complexity and efficiency of a model when processing data.

Table4:

**Table 4. Comparative test results.**

| Model | mAP @0.5  （%） | mAP @0.5:0.95  （%） | Precision  （%） | Recall  （%） | Parameters  （M) | FPS | GFLOPS |
| --- | --- | --- | --- | --- | --- | --- | --- |
| RT-DETR | 89.9 | 53.5 | 89.2 | 85.4 | 31.3 | 90.0 | 103.5 |
| YOLOv5n | 88.1 | 51.0 | 79.6 | 83.9 | 2.6 | **125.1** | 7.1 |
| YOLOv6n | 85.1 | 47.5 | 74.2 | 81.5 | 4.2 | 85.3 | 9.8 |
| YOLOv7-PM | 90.1 | 49.5 | 89.2 | 84.1 | 71.5 | 81.9 | 189 |
| PiX2PiX  +YOLOv7-PM | 91.8 | 52.5 | **92.4** | 83.9 | 92.3 | 75.6 | 231.2 |
| YOLOv9-c | 86.9 | 47.4 | 88.0 | 76.9 | 25.3 | 35.0 | 103.6 |
| YOLOv11n | 91.1 | 56.3 | 84.3 | 83.9 | 2.6 | 82.0 | 6.3 |
| YOLOv12n | 90.5 | 54.9 | 87.5 | 83.0 | **2.5** | 99.1 | **5.8** |
| YOLOv8n  -Baseline | 89.8 | 54.2 | 84.9 | 82.9 | 3.2 | 89.2 | 8.1 |
| **S2DB-mmWave**  **YOLOv8n** | **93.1** | **55.8** | 89.4 | **90.6** | 3.2 | 89.3 | 8.1 |

Line382-Line388:

The S2DB-mmWave YOLOv8n model achieves high accuracy with a computational complexity of 8.1 GFLOPs, positioning it in the lower-middle range among comparative baseline modes. This indicates well-preserved feature extraction capabilities without substantial computational overhead. The model attains a competitively high FPS, demonstrating real-time processing efficiency. Furthermore, its efficient architecture design ensurs favorable scalability and hardware compatibility, enabling deployment across diverse operational scenarios.

Table5:

**Table 5. Results of ablation experiments.**

| NO. | BiFPN | Dysample | Sim Backbone | mAP  @0.5  (%) | mAP  @0.5:0.95  (%) | Precision  (%) | Recall  (%) | FPS | GFLOPs |
| --- | --- | --- | --- | --- | --- | --- | --- | --- | --- |
| 1 | × | × | × | 89.8 | 54.2 | 84.9 | 82.9 | **89.3** | **8.1** |
| 2 | √ | × | × | 91.6 | 53.4 | 88.3 | 85.3 | 48.1 | 8.1 |
| 3 | × | √ | × | 90.3 | 55.1 | 83.8 | 86.3 | 89.3 | 8.1 |
| 4 | × | × | √ | 91.3 | 54 | 86.9 | 84.4 | 87.7 | 8.1 |
| 5 | √ | √ | × | 92.3 | 54.5 | 86.6 | 89.1 | 87.0 | 8.1 |
| 6 | √ | √ | √ | **93.1** | **55.8** | **89.4** | **90.6** | 89.3 | 8.1 |

Line422-Line423:

but also maintains the FPS and GFLOPs, ensuring efficiency and performance.

***5). Consider adding a dedicated subsection discussing the limitations of the current approach, such as potential challenges in highly cluttered environments or varying weather conditions, and how these might be addressed in future work.***

**Response:** Thank you for your careful work and kind comments. We fully appreciate and acknowledge your suggestions. In line with the overall structure of the manuscript, we have added an analysis of the limitations of the current method and the proposed solutions for future work in the conclusion section. We believe these additions will provide a more comprehensive perspective and address the concerns raised effectively.

Revised Section:

Line477-Line483:

(3) multi-object detection directly on millimeter-wave radar heatmaps still faces significant limitations. Radar heatmaps often suffer from low spatial resolution, severe noise, and object occlusion in dense scenes, making it difficult to distinguish overlapping targets. These challenges lead to degraded detection performance, particularly in scenarios with multiple closely spaced or weak-reflecting objects. Therefore, improving heatmap quality, enhancing instance-level feature separation, and incorporating cross-modal priors remain critical directions for future research.

**Finally, we extend our sincere thanks once again for your insightful comments and constructive suggestions on our manuscript. Your feedback has been invaluable in helping us enhance the quality and clarity of our work. We are also deeply grateful for your recognition of the significance and contributions of our research. Your support and guidance are truly appreciated.**

We believe these revisions have substantially improved the manuscript's quality and technical soundness. Should further clarifications be required, we remain available to provide additional information.

Thank you once again for your guidance in advancing this work.

Best regards!

Sincerely,

Yajing Yuan, Dr.

Xuchang University

E-mail: yyjxcu@126.com
